# Research on Polarization Modulation of Electro-Optical Crystals for 3D Imaging Reconstruction

**DOI:** 10.3390/mi15081023

**Published:** 2024-08-11

**Authors:** Houpeng Sun, Yingchun Li, Huichao Guo, Chenglong Luan, Laixian Zhang, Haijing Zheng, Youchen Fan

**Affiliations:** 1 Graduate School, Space Engineering University, Beijing 101416, China; sunhoupeng@hgd.edu.cn (H.S.); lclzdfcs@hgd.edu.cn (C.L.); 2 Space Engineering University, Beijing 101416, China; zhanglaixian@126.com (L.Z.); 3120120251@bit.edu.cn (H.Z.); love193777@sina.com (Y.F.)

**Keywords:** 3D imaging reconstruction, electro-optical crystals, polarization modulation, high-resolution

## Abstract

A method for enhancing the resolution of 3D imaging reconstruction by employing the polarization modulation of electro-optical crystals is proposed. This technique utilizes two polarizers oriented perpendicular to each other along with an electro-optical modulation crystal to achieve high repetition frequency and narrow pulse width gating. By varying the modulation time series of the electro-optical crystal, three-dimensional gray images of the laser at different distances are acquired, and the three-dimensional information of the target is reconstructed using the range energy recovery algorithm. This 3D imaging system can be implemented with large area detectors, independent of the an Intensified Charge-Coupled Device (ICCD) manufacturing process, resulting in improved lateral resolution. Experimental results demonstrate that when imaging a target at the distance of 20 m, the lateral resolution within the region of interest is 2560 × 2160, with a root mean square error of 3.2 cm.

## 1. Introduction

Laser 3D imaging represents an active imaging detection technology unaffected by ambient light and atmospheric conditions, enabling rapid and comprehensive acquisition of target size and structural information [1,2,3,4,5]. This technology finds diverse applications in target observation and recognition [6,7,8,9,10,11]. The area array detector is used as an imaging device in laser three-dimensional imaging technology, which can obtain target information in a single imaging. It has the advantages of fast imaging and high resolution [1,12,13]. The traditional area array laser 3D imaging technology selects an Intensified Charge-Coupled Device (ICCD) with a gating function as the imaging detector device. However, ICCD cameras need to go through the conversion of “optical–electrical–optical–electrical” signals during the imaging process, which leads to a low quantum efficiency of the camera, usually less than 50%, which reduces the imaging quality [14]. In recent years, laser 3D imaging based on polarization modulation has gradually become a research hotspot for area array laser 3D imaging by using large-area array and high-sensitivity industrial cameras (EMCCD: Electron Multiplying Charge-Coupled Device, high-performance CCD, etc.) instead of an ICCD as detectors [15,16,17,18,19]. Polarization modulation refers to a way to modulate a laser to change the polarization state of the laser. Because the detector selected for polarization-modulated 3D imaging reconstruction does not have the gating function or the global shutter gate width is too wide (generally in the order of ms), it is challenging to achieve high-precision distance resolution. Therefore, it is necessary to place an electro-optical crystal at the front end of the detector, which means that a voltage is applied to both ends of the crystal to make it an electro-optical modulator. The electro-optical effect of the crystal is used to realize the gating function of high repetition frequency and narrow pulse width.

In 2016, Sungeun Jo, a scholar at the Korean Academy of Science and Technology, developed a polarization-modulated 3D imaging reconstruction system based on a split focal plane [20]. This innovative system incorporated four micro polarizer arrays aligned in the polarization direction in front of a detector. It calculated the distance information of the target using the polarization data from the laser echo, achieving an impressive lateral resolution of 200 × 200. In 2018, Chen Zhen et al. from the Chinese Academy of Sciences selected an EMCCD as an imaging detector [16] and used a KDP crystal to construct a polarization-modulated 3D imaging reconstruction system. The system has a detector lateral resolution of 1024 × 1024 and a modulation gate width of 0.32 μs, with a range resolution of around 1 m when imaging targets up to 960 m away. In 2020, Song of the University of Aerospace Engineering selected a KTN crystal as the electro-optical modulator [21] to achieve polarization-modulated 3D imaging reconstruction with a large field of view of 20°, with a distance error of 4.8 cm and a distance accuracy of 4.4 cm at 15 m. However, the growth of the KTN crystal is poor, and the clearance diameter of the crystal is only 5 mm, which will waste a lot of target echo information and reduce the imaging quality. In 2023, our group conducted a preliminary study on 3D imaging reconstruction based on electro-optical crystal modulation, and we analyzed the source of the non-uniformity error caused by an electro-optical crystal and calculated the mathematical expression of the non-uniformity of the crystal. Based on the above analysis, a compensation method was proposed to improve the imaging accuracy [22].

At present, 3D imaging reconstruction technology based on polarization modulation uses electro-optical crystals to modulate the laser echo, carrying the target information. It uses the correspondence between the electro-optical crystal modulation state and the flight time to calculate the target distance information, which requires linear modulation of the crystal [23]. However, crystals with high stability and good electro-optical performance, such as KDP and LN crystals, have half-wave voltages of thousands of volts, and the crystals will introduce huge electro-optical modulation errors in the process of high-voltage linear modulation, which will reduce the imaging accuracy of the system. Therefore, in this paper, pulse modulation is first used to realize the two modulation states of electro-optical crystals, “on” and “off.” Then, the detector is used to obtain the target laser gray image with a specific distance and depth of field. Finally, the relevant algorithm is used to recover the distance information of the target to achieve high-resolution laser three-dimensional imaging.

## 2. Comparison of the Related Research Works

Three-dimensional imaging reconstruction is a popular field that has been studied by many scholars; in this section, we will compare previous related research works. In order to distinguish this research from earlier contributions and studies, it is necessary to elaborate from two aspects: one is the difference between the work in this paper and the method of imaging using an ICCD, and the other is the difference between the work of this paper and the method of imaging using an EMCCD. Below we will elaborate on the differences between the three separately.

The imaging camera used in this paper is an SCMOS (edge 5.5, produced by PCO in Kelheim, Germany), and we compare the performance of another ICCD (PI-MAX4: 1024i, manufactured by Teledyne Corporation, Thousand Oaks, CA, USA) camera in the same price range, as shown in Table 1.

In Table 1, SCMOS cameras in the same price range perform better than the ICCD and therefore also have higher imaging potential. In Table 1, the SCMOS has a high quantum efficiency (80%) and low readout noise (<1.0 e^−^), allowing it to capture sharper images in low light. Furthermore, the SCMOS has a high sensitivity and is suitable for applications that require a high signal-to-noise ratio. The ICCD, on the other hand, has a low quantum efficiency (25%), and the ICCD uses electronic enhancement technology to amplify low-intensity light signals to improve imaging sensitivity. The ICCD amplifies the optical signal while simultaneously amplifying the noise, thus introducing imaging errors. In addition, due to the difference in manufacturing process, the SCMOS (pixel counts: 2560 × 2160) also has a higher pixel count than the ICCD (pixel counts: 1024 × 1024), which is more conducive to high-resolution imaging. However, the ICCD has its own shutter and has the function of gating a target at a specific distance, and the SCMOS imaging performance is high, but it does not have the gating function, so the electro-optical effect of the crystal is used to realize the gating function of the SCMOS. In the laboratory experiment, the root mean square error of the SCMOS imaging at the distance of 20 m can reach 3.2 cm, and the root mean square error of the ICCD is around 5 cm under the same imaging environment.

**Figure 1:**
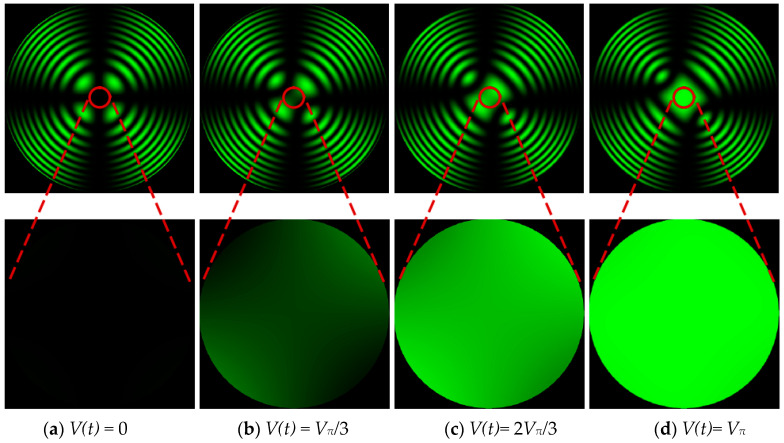
Schematic diagram of the modulation process of a crystal by EMCCD imaging.

Some scholars use an EMCCD for 3D imaging restoration studies. Both our imaging method and the EMCCD imaging method use the electro-optical effect of the crystal to achieve the 3D imaging function of the target. However, the imaging principle of the two methods is different, so the modulation of the crystal is also different. Figure 1 illustrates the modulation process of a crystal using an EMCCD.

The EMCCD imaging method uses an EMCCD to obtain the polarization light intensity information of the target by linear modulation of the crystal, and calculates the distance information of the target by solving the relationship between the polarization state of the crystal modulation and the time of flight. As can be seen from Figure 1, the modulation uniformity of the crystal is good at *V*(*t*) = 0 and *V*(*t*) = *V_π_* during modulation, while the uniformity of the crystal is poor in other modulation processes. Due to the inhomogeneity of the crystal, distance errors in imaging are introduced. Therefore, in this paper, the modulation mode of the crystal is changed from linear modulation to pulse modulation, and the two modulation states of *V*(*t*) = 0 and *V*(*t*) = *V_π_* of crystal modulation are used to gate the target, and then the algorithm introduced in Section 3.2 of the manuscript is used to recover the distance information of the target, which can reduce the modulation error of the crystal and improve the imaging accuracy.

## 3. Imaging Principles

### 3.1. Electro-Optic Crystal Polarization Modulation Gated Imaging Model

**Figure 2:**
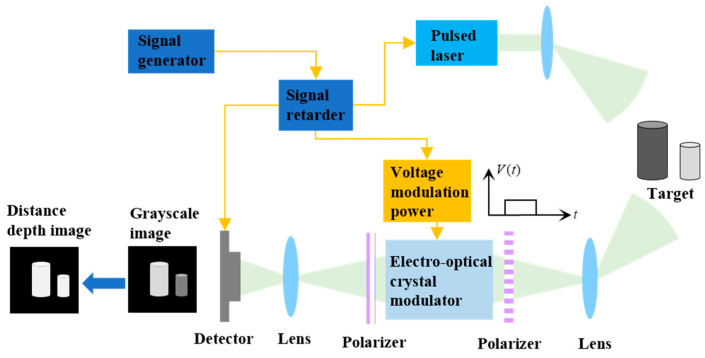
Schematic diagram of 3D imaging reconstruction based on polarization modulation gating of electro-optical crystal.

**Figure 3:**
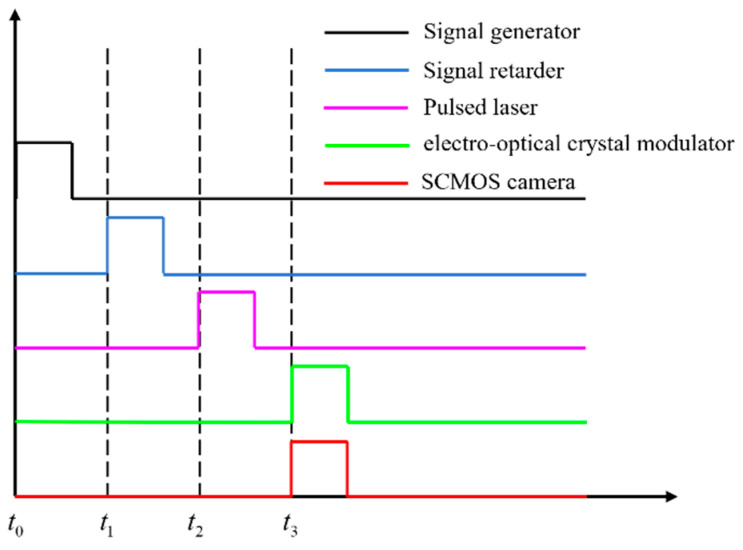
Signal timing diagram.

The 3D imaging reconstruction technology based on electro-optical crystal polarization modulation gating requires strict control of the timing signal. Its imaging principle is shown in Figure 2, and the timing signal relationship is shown in Figure 3. The imaging principle can be described as follows: the signal generator emits a signal at time $t_{0}$, and the signal is transmitted to the signal retarder at time $t_{1}$. The signal retarder sends out three signals at $t_{1}$, which trigger the laser, the electro-optical crystal modulator, and the imaging detector, respectively. At the $t_{2}$ moment, the laser receives the trigger signal from the signal retarder and emits one laser pulse, which is homogeneous enough to illuminate the target image. The laser echo carrying the target information is transmitted to the electro-optical crystal at time $t_{3}$. The signal delay device controls the output signal delay time, and the delay signal at $t_{3}$ time triggers the electro-optical crystal modulator, opens the crystal shutter, and realizes the gating function of the target imaging information through the electro-optical crystal polarization modulation. At $t_{3}$, the third signal of the signal retarder triggers the detector to open the global shutter to obtain the target imaging gray image. Finally, the distance and depth image of the target is recovered according to the target imaging grayscale to realize the laser three-dimensional imaging of the target.

### 3.2. Distance Information Solving Principle

**Figure 4:**
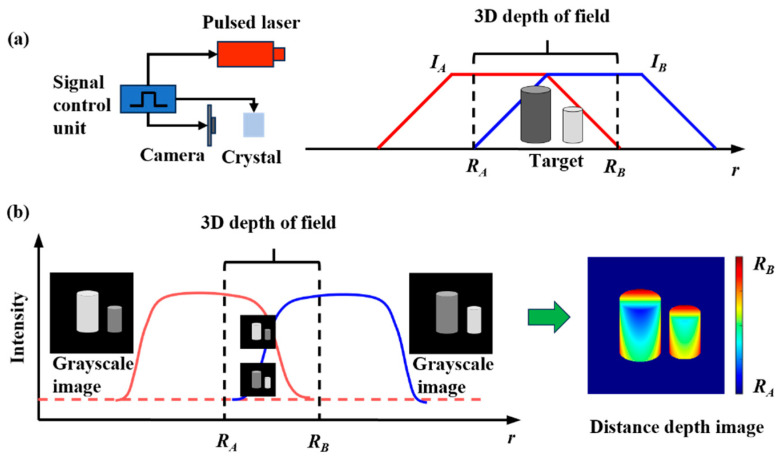
Schematic diagram of crystal polarization-modulated gated 3D imaging: (**a**) 3D imaging process; (**b**) 3D imaging restoration.

There are two ways to solve the distance information of strobe 3D imaging, one is the triangular distance energy solution method, and the other is the trapezoidal distance energy solution method. When using the trapezoidal method to calculate the target distance, there is no need to consider the proportional relationship between the laser pulse width and the crystal shutter width, and the implementation method is more flexible, so the trapezoidal method is used to solve the target distance information. The principle of the trapezoidal method can be simply described as follows: the function of the target echo energy obtained by laser three-dimensional imaging and the distance of the target is approximately trapezoidal, and the target energy is displayed in the form of gray value in the image, so the distance information of the target can be solved through the gray value of the image. Figure 4 shows a schematic diagram of 3D imaging of a crystal polarization-modulated gated laser. In Figure 4a, a pulsed laser emits a laser light to illuminate the target surface. Two raw grayscale images *I_A_* and *I_B_* with trapezoidal range intensity profiles were acquired by the camera. In Figure 4b, the distance range *R_A_*–*R_B_* of the target is recovered from the two grayscale images of the target acquired.

In one imaging cycle of Figure 4, the energy of the echo signal scattered by the target detected by the 3D imaging detector can be expressed as follows:

(1) $$R_{N}(z)=P_{\det}(t)*G_{N}(t)=\int_{0}^{+\infty }P_{\det}(t)\cdot G_{N}(t-\frac{2z}{c})dt$$

**Figure 5:**
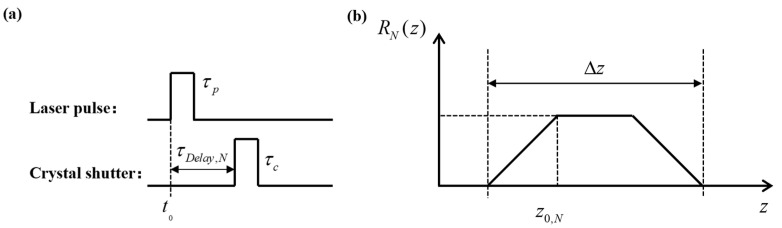
Three-dimensional imaging distance–energy diagram: (**a**) laser pulse and crystal shutter; (**b**) the relationship between the echo energy and the target distance.

$R_{N}(z)$ is the target energy at z of the detector detection range; $P_{\det}(t)$ is the laser pulse energy; *c* is the speed of light; and $G_{N}(t)$ is the waveform expression of the electro-optical modulation crystal shutter. When the shutter is opened, $G_{N}(t)=1$ and the rest of the time is 0. When the target distance *z* changes, the received echo energy $R_{N}(z)$ changes accordingly, so that the relationship between the echo energy $R_{N}(z)$ and the target distance *z* is established, which is called the three-dimensional imaging distance energy relationship, and Figure 5 is the timing diagram of the laser pulse and the shutter for an imaging period.

In Figure 5, the laser pulse width is $\tau_{p}$, the shutter time is $\tau_{c}$, *c* is the speed of light, and Δz is the depth of field of a slice image: $\Delta z=c\cdot (\tau_{p}+\tau_{c})/2$. $z_{0,N}$ is the reference distance, which can be expressed as follows: $z_{0,N}=c\cdot \tau_{Delay,N}/2$.

Usually, imaging devices operate in the linear region within their dynamic range, where the signal energy is approximately proportional to the image grayscale value. Therefore, it is possible to establish a relationship between the grayscale values of laser images and the distance of the target. By observing the distance–energy correlation curve, it can be observed that the grayscale values do not correspond one-to-one with the target distance *z*, indicating that the distance information of the target cannot be obtained from a single distance slice image. Therefore, it is necessary to use multiple distance slice images to solve the problem of the grayscale values of the image not corresponding one-to-one with the distance. As shown in Figure 5, the shaded area marked can be utilized to obtain the target distance information using two distance slice images.

**Figure 6:**
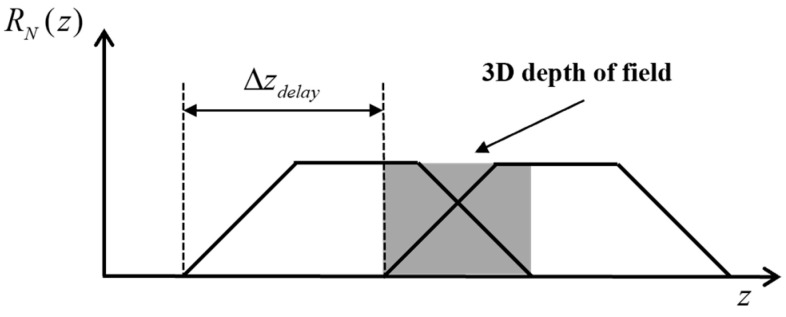
Schematic diagram of the energy of two echo signals.

For two distance slice images with a time-lapse step of Δτ, the step size of the two distance slice images is defined as follows: $\Delta z_{delay}=c\cdot \Delta \tau /2$. Figure 6 shows the energy distribution of the two echo signals under ideal conditions. For slice images with different distance delay times $\tau_{Delay,N}$, the imaging distance from the slice image is different, and the imaging is affected by factors such as laser beam divergence and atmospheric attenuation. In addition, the gray value of the target in the range-gated image is affected by the distance between the target and the imaging system, the reflectivity of the target itself, and other factors. Therefore, it is still a problem to directly use the gray value of the image to calculate the distance information of the target. In order to exclude the influence of the above factors, the relationship between the echo energy received by the distance gating gate and the target distance *z*, that is, the relationship between the pixel gray value of the distance gating image and the target distance *z*, needs to be normalized: the function $I_{N}(z)$ is defined, for the target point at the distance *z*, by the ratio of the actual echo energy obtained under specific lighting conditions to the maximum echo energy that can be obtained under ideal lighting conditions, so that the normalization of the echo signal energy can be realized, and the expression form of the function $I_{N}(z)$ is as follows:

(2) $$I_{N}(z)=\frac{Q_{N}(t)}{\max(Q_{N})}=\frac{L}{L_{\max}}$$

where $Q_{N}(t)$ is the actual echo energy received, $\max(Q_{N})$ is the maximum echo energy that can be received, *L* is the pixel gray value of the target in the slice image at the distance *z*, $L_{\max}$ is the maximum gray value of the distance slice image, and the maximum value of the $I_{N}(z)$ function is 1.

**Figure 7:**
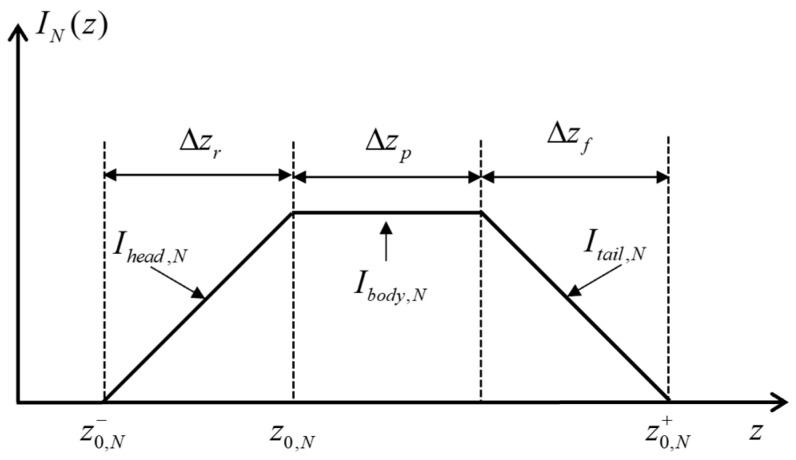
Distance grayscale curve of a trapezoid.

The waveform of the laser pulse width and shutter response will work together to determine the shape of the distance grayscale curve. Figure 7 shows the shape of the distance grayscale curve of a trapezoid.

In Figure 7, $z_{0,N}^{-}$ and $z_{0,N}^{+}$ represent the minimum and maximum distance information contained in the distance slice image, respectively. The $I_{N}(z)$ functions in the corresponding sub-intervals of $\Delta z_{r}$, $\Delta z_{p}$, and $\Delta z_{f}$ are called “head” signals, “body” signals, and “tail” signals, which are denoted as $I_{head,N}$, $I_{body,N}$, and $I_{tail,N}$, respectively. The values of $\Delta z_{r}$, $\Delta z_{p}$, and $\Delta z_{f}$ are the depth of field of the corresponding sub-section.

Any distance *r* in the effective distance interval can be solved for, and the calculation formula is as follows:

(3) $$r=\left\{\begin{array}{cc} z_{0,N}+\frac{I_{head,N+1}\left(z\right)}{I_{body,N}}\cdot \frac{c\cdot \tau_{p}}{2} & ,z\in \left[z_{0,N},z_{0,N}+c\cdot \tau_{p}/2\right]\\ z_{0,N+1}+\left[1-\frac{I_{tail,N}\left(z\right)}{I_{body,N+1}}\right]\cdot \frac{c\cdot \tau_{p}}{2} & ,z\in \left(z_{0,N}+c\cdot \tau_{p}/2,z_{0,N}+c\cdot \tau_{p}\right] \end{array}\right.$$

After the gray image of the target is obtained by using the crystal polarization modulation range gating imaging system, the distance of the target is recovered by using the above calculation formula, and the distance depth map of the target is obtained.

## 4. Analysis of Polarization Modulation Characteristics of Electro-Optical Crystals

In the 3D imaging reconstruction of electro-optical crystal polarization modulation, the electro-optical crystal provides the detector with a high repetition frequency and narrow pulse width gating function, and the polarization modulation characteristics of the electro-optical crystal affect the 3D imaging reconstruction quality. The lithium niobate (LiNbO_3_, LN) crystal selected in this paper was used for electro-optical polarization modulation research, and the LN crystal belongs to the C3v − 3 m crystal class, which has the characteristics of a large electro-optical coefficient and low half-wave voltage and is a crystal material with excellent electro-optical properties. In this paper, we will analyze the electro-optical modulation characteristics of crystals under arbitrary incidence conditions according to the refractive index ellipsoid theory and explore the effective field of view of electro-optical crystal modulation in 3D imaging reconstruction systems.

In the absence of an external electric field, the refractive index ellipsoidal equation of an LN crystal can be expressed as follows:

(4) $$\frac{X^{2}}{{n_{o}}^{2}}+\frac{Y^{2}}{{n_{o}}^{2}}+\frac{Z^{2}}{{n_{e}}^{2}}=1$$

where *X*, *Y*, and *Z* are the main refractive index axes of the crystal and $n_{o}$ and $n_{e}$ are the refractive indices of ordinary light and abnormal light, respectively. Now, the crystal is uniaxial, and the optical axis of the crystal is along the *Z*-axis.

When the LN crystal is used for electro-optical modulation, if the direction of the light is along the optical axis (*Z* axis), and if the direction of the external electric field is also along the optical axis, the two refracted rays corresponding to the perpendicular incident light have the same refractive index. There is no phase difference in the propagation process of the crystal, so it is not easy to achieve a practical modulation effect. Therefore, when the direction of light is along the *Z*-axis, the direction of the electric field should be along the *X*-axis or *Y*-axis, and the modulation effect of the two methods is the same. When an electric field is applied along the *X*-axis, the refractive index ellipsoidal coordinate system will rotate 45° about the *Z*-axis to become an *O*-*X*’*Y*’*Z*’ coordinate system.

**Figure 8:**
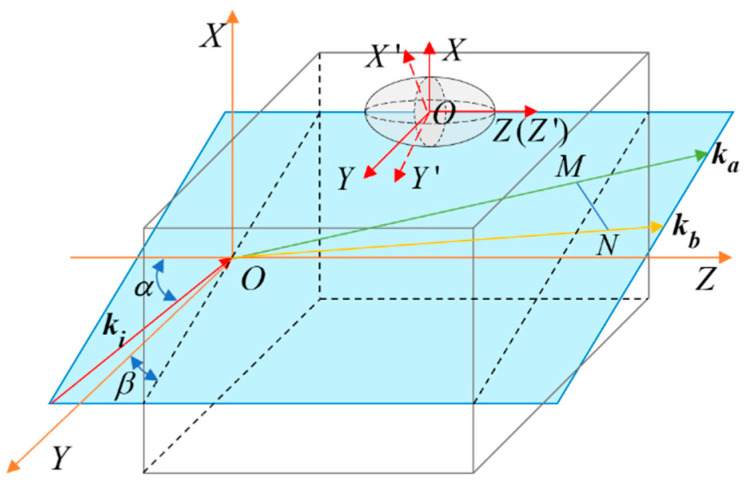
The propagation of light in an electro-optical crystal.

The propagation of light in the crystal is shown in Figure 8. In the *O*-*XYZ* coordinate system, the light vector $k_{i}$ enters the crystal at the angle of incidence α and the azimuth angle β. Due to the birefringence effect, $k_{i}$ is refracted on the surface of the crystal into two light vectors, $k_{a}$ and $k_{b}$. The intersection points of $k_{a}$ and $k_{b}$ with the exit surface of the crystal are *M* and *N*, respectively. In the 3D coordinate system *O*-*XYZ*, the incident light $k_{i}$ can be expressed as $k_{i}={\left(\sin\alpha \cos\beta ,\sin\alpha \sin\beta ,\cos\alpha \right)}^{T}$.

Since the incident light vector $k_{i}$ enters the crystal, and the birefringence effect will produce two light vectors $k_{a}$ and $k_{b}$ in the crystal, we have analyzed in Reference [24] that the average value k of the two refracted light vectors can be used as the refracted light vector for study. The influence of this simplified research method on the modulation performance of the electro-optical crystal is negligible. When the direction of light is along the optical axis, the phase delay caused by the electro-optical effect can be expressed as follows:

(5) $$\Gamma =\frac{2\pi }{\lambda }n_{o}^{3}\gamma_{22}\frac{V}{d}L$$

where V is the voltage applied at both ends of the crystal, and d is the thickness of the crystal in the direction of the external electric field. When Γ=π, $V=\frac{\lambda }{2n_{o}^{3}\gamma_{22}}\frac{d}{L}$, it is called a half-wave voltage $V_{\pi }$.

Calculating the interference light intensity requires the phase difference θ(α,β) of the birefringent ray and the polarization direction ϕ(α,β) of the refracted light. The expression for phase difference θ(α,β) is as follows:

(6) $$\begin{array}{c} \theta (\alpha ,\beta )=\frac{2\pi }{\lambda }(\frac{L}{\cos\varphi_{a}}n_{a}-\frac{L}{\cos\varphi_{b}}n_{b})\\ \approx \frac{2\pi L(n_{a}-n_{b})}{\lambda \cos\varphi } \end{array}$$

where ϕ(α,β) is the angle of refraction of the refracted ray, $\varphi (\alpha ,\beta )=\arcsin(\frac{\sin\alpha }{n_{a}+n_{b}})$.

**Figure 9:**
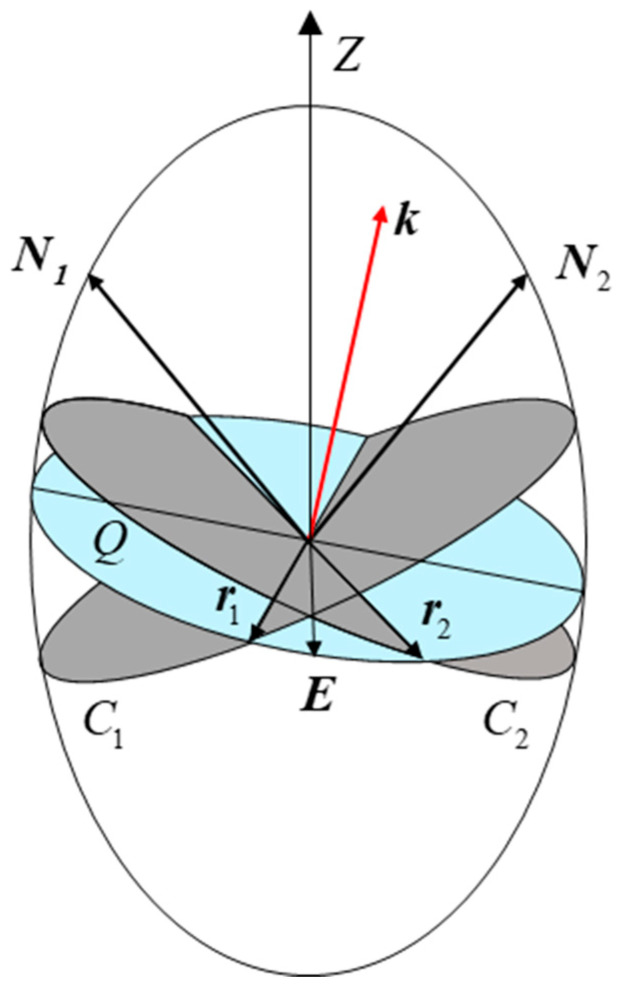
SRefractive index ellipsoid.

To find the polarization direction of the refracted ray, ϕ(α,β), requires the help of a refractive index ellipsoid, which is shown in Figure 9. An ellipsoid has two circular cross-sections, C_1_ and C_2_, passing through the center of the sphere, and the normals of these two cross-sections, *N*_1_ and *N*_2_, are called the optical axis of the crystal; *N*_1_ and *N*_2_ are in the *X*’*OZ*’ plane, and in the *O*-*XYZ* coordinate system, the unit typical of the optical axis surface is $\rho ={\left(\frac{\sqrt{2}}{2},\frac{\sqrt{2}}{2},0\right)}^{T}$. For a ray k, propagating in a non-optical axis direction, the center of the super refractive index ellipsoid is a plane *Q* perpendicular to k, and its cross-section with the ellipsoid is an ellipse. The plane *Q* intersects the circle C_1_ and C_2_ at $r_{1}$ and $r_{2}$, which are of equal length, so the principal axis direction of the ellipse *Q* is the direction of the angular bisector of $r_{1}$ and $r_{2}$, which is also the direction of polarization E of the ray k.

The three-dimensional coordinates of the unit direction vectors of the optical axis *N*_1_ and *N*_2_ are $N_{1}=(\cos\eta ,0,\sin\eta )$ and $N_{2}=(\cos\eta ,0,-\sin\eta )$, respectively. η is the angle between the optical axis and the *Z* axis, and the expression of η is given directly:

(7) $$\eta =\arctan(\frac{n_{1}}{n_{3}}\sqrt{\frac{{n_{2}}^{2}-{n_{3}}^{2}}{{n_{1}}^{2}-{n_{2}}^{2}}})$$

Since $r_{1}$ is perpendicular to k and *N*_1_, $r_{1}$ can be found, and similarly, $r_{2}$ can be found

(8) $$\left\{\begin{array}{c} r_{1}=\frac{k\times N_{1}}{\left|k\times N_{1}\right|}\\ r_{2}=\frac{k\times N_{2}}{\left|k\times N_{2}\right|} \end{array}\right.$$

The direction of polarization of the light rays is $E=\frac{1}{2}(r_{1}+r_{2})$. The angle between the polarization direction and the optical axis surface can be expressed as follows:

(9) $$\phi (\alpha ,\beta )=\arcsin(\frac{E\cdot \rho }{\left|E\right|\left|\rho \right|})$$

Knowing the phase difference *θ*(*α*,*β*) and the polarization direction *ϕ*(*α*,*β*), the light intensity formula can be expressed as follows:

(10) $$I(\alpha ,\beta )=\sin^{2}[2\phi (\alpha ,\beta )]\sin^{2}[\frac{\theta (\alpha ,\beta )}{2}]$$

**Figure 10:**
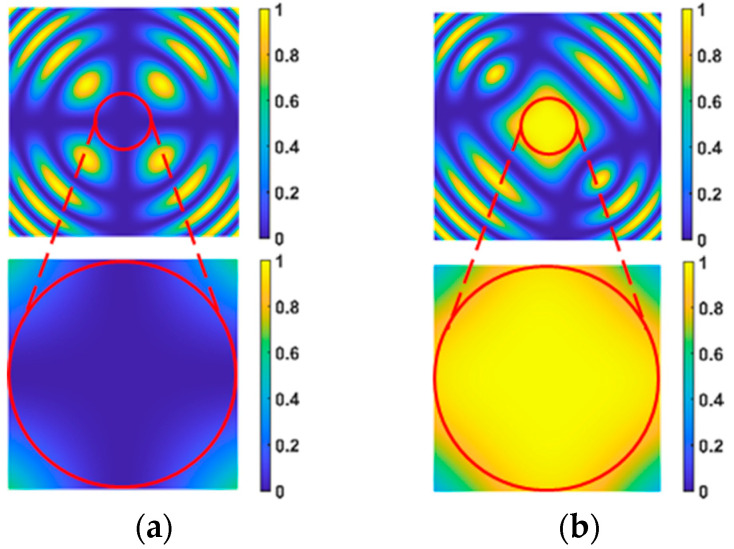
Polarization modulation of electro-optical crystal light intensity distribution. (**a**) the light intensity distribution of the electro-optical crystal in the “off” state; (**b**) the light intensity distribution of the electro-optical crystal in the “on” state.

According to Equation (8), the light intensity distribution under electro-optical crystal modulation can be calculated, and the result is shown in Figure 10.

As shown in Figure 10, Figure 10a shows the light intensity distribution of the electro-optical crystal in the “off” state, the lower part of Figure 10a is the magnification of the central region of the upper half, Figure 10b is the light intensity distribution of the electro-optical crystal in the “on” state, and the lower part of Figure 10b is the magnification of the central region of the upper half.

To achieve high-precision electro-optical crystal polarization modulation, the modulation effect is measured according to the light intensity, brightness, and uniformity of the electro-optical crystal modulation, and the field of view of electro-optical crystal polarization modulation is determined. LN was selected as the electro-optical modulation crystal, and the clear aperture of the crystal was 9 mm × 9 mm, and the modulation length was 18.8 mm. Assuming that the brightest light intensity of the central field of view is 1 during the crystal polarization modulation when the average light intensity of the modulation region is greater than 0.95, and the lowest light intensity is less than 0.9, it is considered that the polarization modulation light intensity of the electro-optical crystal has good uniformity. The field of view of the electro-optical crystal polarization modulation is around 0.5 degrees when the above conditions are met.

**Figure 11:**
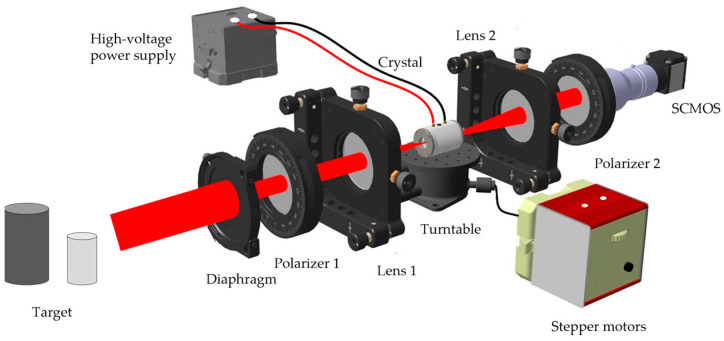
Schematic diagram of crystal polarization modulation.

Figure 11 is a schematic diagram of crystal polarization modulation. In Figure 11, the laser echo scattered by the target passes through the diaphragm and enters a crystal polarization modulator, which consists of two polarizers with perpendicular polarization directions, two lenses, and a crystal. The high-voltage power supply provides voltage to the crystal, the stepper motor drives the turntable, and the crystal placed on the turntable can achieve high-precision three-dimensional angle control. The laser echo carrying the target information is modulated by a modulator and then received by the Scientific Complementary Metal-Oxide Semiconductor (SCMOS) camera.

According to the polarization-modulated light intensity distribution of the electro-optical crystal, the central region of the electro-optical modulation interference pattern can be used to realize the three-dimensional laser imaging of polarization modulation, which requires the returned laser echo to be in the middle region of the crystal interference pattern. For short-range targets, the field of view of 3D imaging reconstruction based on electro-optical crystal polarization modulation will be limited to a slight angle. However, for long-distance targets, the divergence angle of the laser echo is slight, and the energy of the laser device will become a significant limitation of the 3D imaging of this system. Therefore, it is necessary to optimize the reasonable balance between the field of view of electro-optical crystal modulation and the laser energy to build a 3D imaging reconstruction system with the best performance.

## 5. Experimental Prototype Construction

### 5.1. Prototype Construction

**Figure 12:**
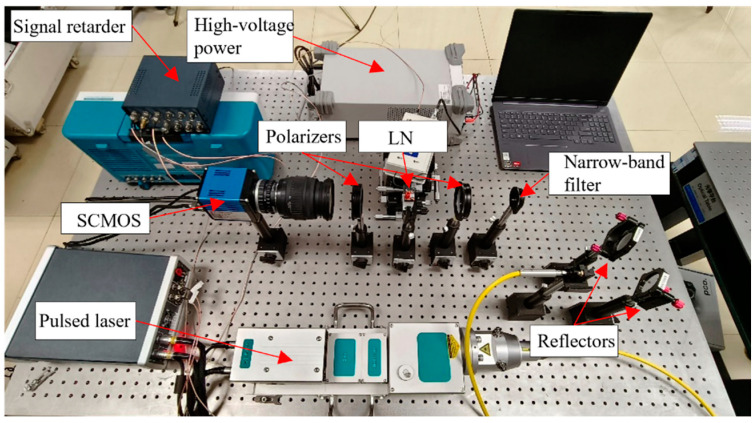
The prototype of the 3D imaging system is based on electro-optical crystal polarization modulation.

According to the principle of electro-optical crystal polarization modulation and 3D imaging, we have built a prototype of a 3D imaging reconstruction system of electro-optical crystal polarization modulation gating in the laboratory, and the experimental prototype is shown in Figure 12. The prototype consists of laser emission, electro-optical crystal polarization modulation, timing signal control, and laser echo reception. The laser emission part is composed of the following devices: a pulsed xenon lamp-pumped Nd; a YAG Q-switched laser, which is used to provide uniform illumination for the target of the laser emitted; and a microlens array, which is placed at the front end of the laser to collimate and homogenize the laser beam emitted by the laser, and the collimated and homogenized laser uses two reflectors to control the illumination angle of the laser beam so that it can accurately irradiate the target center area. After the laser is irradiated on the target surface, the laser echo first passes through a narrow-band filter to filter out the background stray light. Then, the laser echo enters the polarization modulation part of the electro-optical crystal of the prototype. The electro-optical crystal modulation part is composed of a high-voltage modulation power supply, a lithium niobate crystal, and polarizers placed on both sides of the crystal that are perpendicular to each other’s polarization directions, and the high-voltage power supply provides a modulation voltage for the crystal. If the crystal optical switch is turned off under the action of the driving voltage, the optical switch blocks the pulsed laser, and the detector cannot receive the laser echo; if the crystal optical switch is open under the action of the impulse voltage at this time, the laser pulse echo is received by the detector, and the detector selected for the experiment is the SCMOS (Scientific Complementary Metal-Oxide Semiconductor); the SCMOS is a scientific CMOS detector, which is a high-precision, high-sensitivity, and high-dynamic range detector, which is often used for high-precision imaging. After the SCMOS receives the laser echo, the prototype completes a gated imaging. Since our imaging system is an active imaging method, it can be used around the clock, regardless of climatic conditions. Calibration is necessary for the imaging system, and the experiment is strictly in accordance with afront to back order to build the experimental system; after the construction, the experimental system is fine-tuned according to the actual imaging effect to ensure that the system can carry out clear and accurate imaging.

The main parameters for the 3D imaging system based on electro-optical crystal polarization modulation are shown in Table 2.

### 5.2. Imaging Targets

**Figure 13:**
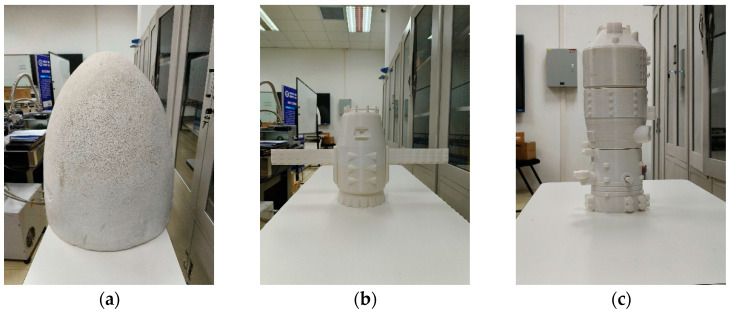
Three-dimensional imaging target. (**a**) semi-ellipsoidal object; (**b**) satellite object; (**c**) space station object.

The electro-optical crystal polarization modulation prototype selects three kinds of targets for laser three-dimensional imaging, and the imaging targets are shown in Figure 13. Figure 13a is a semi-ellipsoidal object with an ellipsoid axis of 70 cm and a minor axis of 55 cm. Figure 13b is a satellite, its size is 56 × 30 × 16 (unit: cm), and Figure 13c is a space station, its size is 40 × 10 × 10 (unit: cm), and the 3D imaging distance of the target is set to 15 m.

### 5.3. Gated Imaging Timing Control

**Figure 14:**
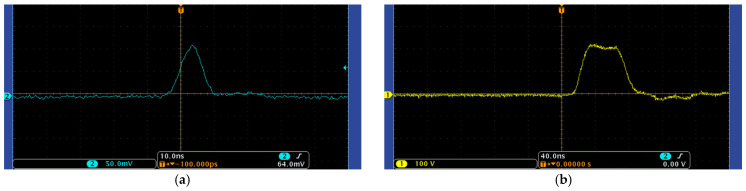
Waveform diagram of the laser pulse and electro-optic-modulated shutter signal. (**a**) Laser pulse waveform; (**b**) The electro-optical crystal modulates the shutter waveform.

To accurately obtain the distance information of the target, it is necessary to strictly control the shutter timing relationship between the laser pulse and the polarization modulation of the electro-optical crystal, and the timing signal control accuracy determines the imaging accuracy. The signal waveform of the laser pulse measured by an oscilloscope and the electro-optical crystal-modulated shutter are shown in Figure 14. As can be seen from Figure 14, the laser pulse waveform is approximately Gaussian with a pulse duration of around 5 ns, and the electro-optical crystal-modulated shutter is approximately trapezoidal with a gate width of around 40 ns and a rise time of around 17 ns.

**Figure 15:**
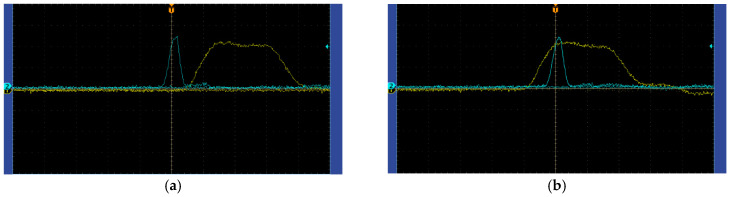
The timing relationship between the laser pulse and electro-optical-modulated shutter signal. (**a**) The laser pulse signal is tangent to the electro-optical crystal-modulated shutter; (**b**) The laser pulse signal enters the electro-optical-modulated shutter completely.

According to the timing relationship between the laser pulse signal and the electro-optical crystal-modulated shutter, the gating information of the shutter can be judged. As shown in Figure 15a, when the returned laser pulse signal is exactly tangent to the electro-optical crystal-modulated shutter signal, the target imaging information happens to enter the detector for imaging. As shown in Figure 15b, when the laser pulse signal completely enters the electro-optical crystal modulation shutter, all the returned target echo information is received by the detector. Through the control of the timing signal by the signal generator, oscilloscope, and signal delay, the detection target can be accurately gated and imaged.

## 6. Experimental Results and Analysis

### 6.1. Two-Dimensional Gating Imaging

**Figure 16:**
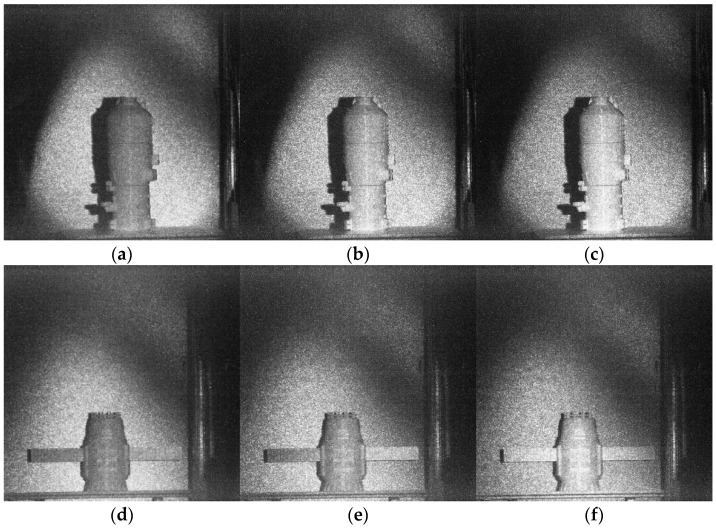

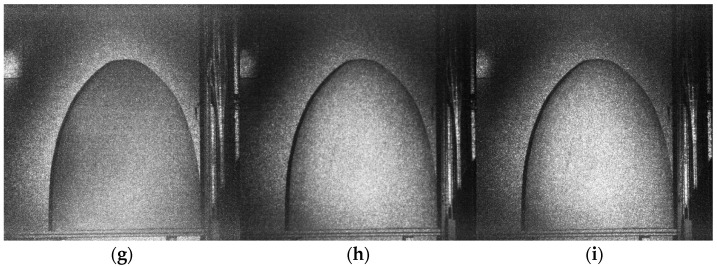
Three-dimensional imaging results. Imaging results of space station (**a**–**c**); imaging results of satellite (**d**–**f**); and imaging results of semi-ellipsoidal object (**g**–**i**).

By controlling the signal retarder to trigger the modulation time of the electro-optical crystal, it is possible to image targets at different distances within the detector’s field of view. Imaging of the semi-ellipsoidal object, satellite, and space station using the prototype built in the laboratory is provided in Figure 13. Figure 16 is the imaging results of the three targets, and for each target three pictures are obtained by the prototype. The imaging uses the rise stage of the gating, in which the rise time is 17 ns, and the delay time difference of each picture is 1 ns. In Figure 16, the bright area in the center of the image is the valid information selected for the modulation of the crystal, and the shadow at the edge of the image is the black streaks due to the optical interference of the crystal cone.

**Figure 17:**
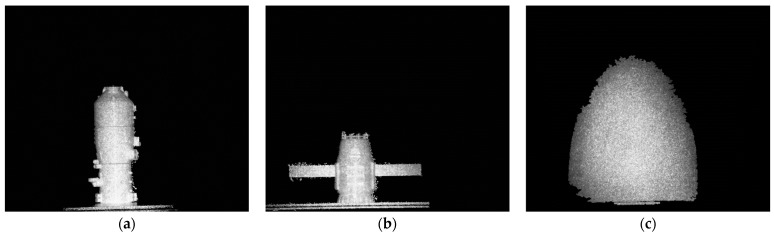
Three-dimensional imaging preprocessing results. (**a**) preprocessing result of the space station object; (**b**) preprocessing result of the satellite object; (**c**) preprocessing result of the semi-ellipsoidal object.

As can be seen from Figure 16, the grayscale image obtained by the prototype of the 3D imaging system has a large amount of speckle noise, which needs to be denoised first. In addition, when using laser grayscale images to recover target distance information, the target extraction operation can be carried out first to reduce the influence of the imaging background on the target recovery algorithm and enhance the accuracy of range recovery. We refer to image denoising and target extraction as preprocessing before range/depth information recovery. In this paper, the DnCNN network in Reference [25] is used for denoising, which can maintain the original details of the image while removing the speckle noise of the image and has good robustness. The preprocessing results of the laser 3D image are shown in Figure 17.

### 6.2. Three-Dimensional Imaging

**Figure 18:**
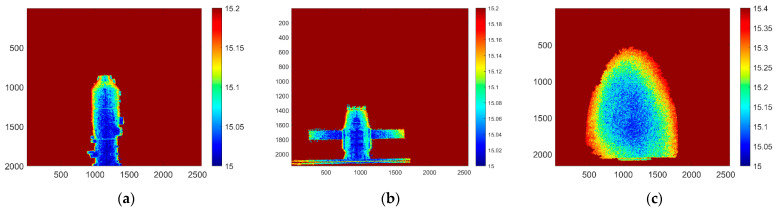
Three-dimensional imaging distance depth image. (**a**) distance depth image of the space station object; (**b**) distance depth image of the satellite object; (**c**) distance depth image of the semi-ellipsoidal object.

After the preprocessing operation of the laser two-dimensional gated grayscale image, the background information of the target is filtered out, and the laser speckle noise is effectively suppressed. The preprocessed image is recovered from the target distance information by using the trapezoidal distance energy algorithm introduced in Section 3.2, and the results are shown in Figure 18.

Figure 18 shows the depth of the range of the three targets after being imaged by polarization-modulated gating of the electro-optical crystal. The imaging distance of these targets is set at 15 m, and the color variation in the graph reflects the distance of the target, and the actual distance of the target is indicated by a color bar on the right. Judging from the imaging recovery results, the details of the target can be clearly resolved, showing a high imaging resolution.

**Figure 19:**
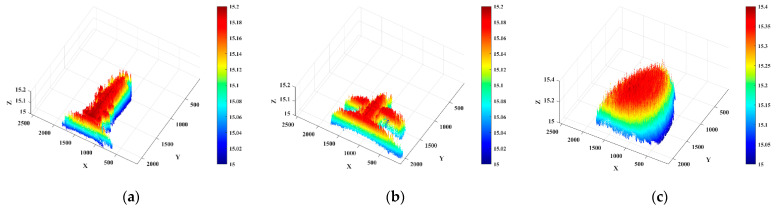
Three-dimensional imaging point cloud image. (**a**) point cloud image of the space station object; (**b**) point cloud image of the satellite object; (**c**) point cloud image of the semi-ellipsoidal object.

To present the three-dimensional information of the target in space more subjectively, the three-dimensional point cloud of the target is recovered by using the distance information of the target in Figure 18, and the result is shown in Figure 19.

Figure 19 shows the laser 3D point cloud data of the target, which can be better observed than the 2D image, which can show the geometric features and contour information of the target. We compare the 3D imaging results in Figure 19 with the true size of the target; the reconstruction depths of the three targets in the z direction are 6.7 cm, 6.9 cm, and 24.3 cm, respectively, and the depths errors of the imaging are 1.7 cm, 1.1 cm, and 3.2 cm, respectively. It has a high imaging accuracy.

### 6.3. Imaging Performance Analysis

**Figure 20:**
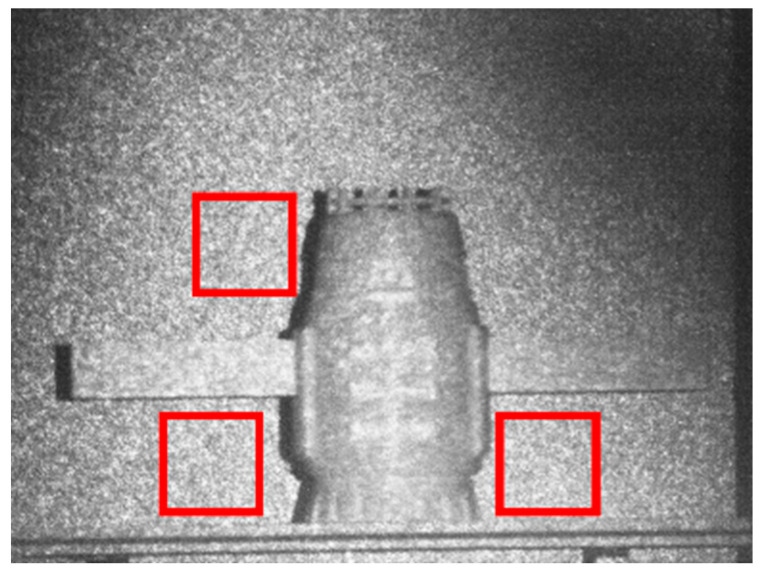
The target area is selected for evaluation measurements; red squares were selected for evaluation; each red square was 100 × 100 pixels.

To evaluate the imaging performance of the prototype, this paper considers the distance resolution of the white wall background during 3D imaging. As shown in Figure 20, three background areas within the imaging field of view were selected for evaluation; each area was 100 × 100 pixels, and the background area was gated for gating imaging. To reduce the influence of random error on the experiment, 100 experiments were averaged as our experimental results.

**Figure 21:**
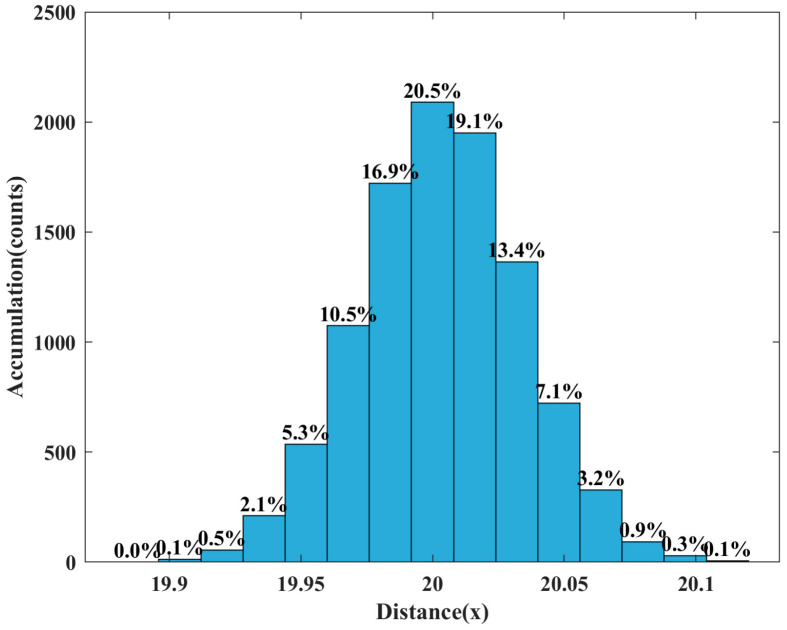
Distance information histogram distribution, the number represents the percentage of distance x.

To visually represent the experiment’s imaging accuracy, the histogram distribution of distance information in the range of 100 × 100 pixels after preprocessing is presented, and the results are shown in Figure 21.

In Figure 21, the distance of 3D imaging is approximately a Gaussian distribution. Statistically, the recovery distance of 3D imaging occupies 92.8% of the 20 ± 0.05 m of the white target wall at the distance of 20 m. The imaging accuracy is higher in general, and some points with a significant ranging error may be caused by the Gaussian speckle noise that has not been removed.

**Figure 22:**
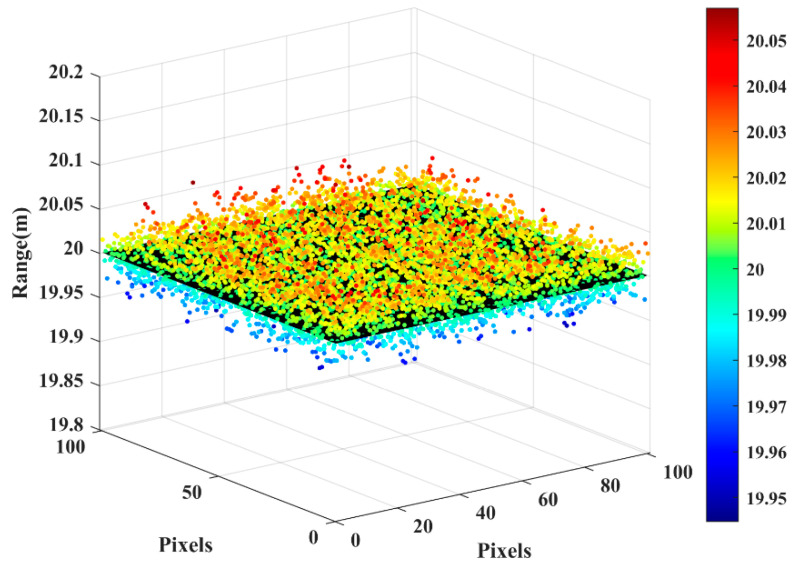
The target area is selected for evaluation measurements.

The root mean square error is used to evaluate the measurement accuracy of the prototype, and the root mean square error can be expressed as follows:

(11) $$RMS=\sqrt{\frac{1}{N}\sum_{i=1}^{N}{\left(x_{i}-m_{i}\right)}^{2}}$$

where *N* is the number of pixels, $x_{i}$ is the measurement distance of the *i*th pixel, and $m_{i}$ is the measurement distance of the *i*th pixel by using the fitting plane. The imaging results of the experiment are shown in Figure 22.

In Figure 22, the black plane is the real distance of the wall background, and the colored dots are the actual distances calculated by the prototype of the 3D imaging system. After calculation, the root mean square error of the 100 × 100-pixel range is 3.2 cm, and the principle prototype has good imaging accuracy.

In this paper, to measure the imaging resolution of the system, we set the flat target to be 20 m away from the detector, so the detection distance is known, compare the recovered distance with the real distance, calculate the imaging resolution of the system, and use the imaging results to analyze the error. In the real world, when imaging a cooperative target, since the size of the cooperative target is known, we can compare the imaging results with the actual size of the target to calculate the imaging resolution and imaging error.

## 7. Conclusions

In this paper, we construct a 3D image reconstruction experiment based on the polarization modulation of electro-optical crystals. The gated imaging of the target is achieved by controlling the laser, electro-optic crystal modulator, and imaging detector through a tight synchronization circuit. After the prototype obtains the laser image, the range depth information of the target is recovered by the range energy recovery algorithm after preprocessing, such as target area extraction and denoising. After analyzing the modulation principle of the electro-optical crystal, the electro-optical crystal used in the system has good uniformity within the field of view of 0.5 degrees. The lateral resolution of the prototype is 2560 × 2160. The target at the distance of 20 m is imaged. The recovery distance of three-dimensional imaging occupies 92.8% in the 20 ± 0.05 m range, and the root mean square error of imaging is around 3.2 cm. The prototype uses an SCMOS camera equipped with an electro-optical-modulated crystal to replace the ICCD detector used in traditional laser 3D imaging, which improves the lateral resolution of the target while maintaining the same range resolution, which provides a new idea for the research of range-gated laser 3D imaging.

Polarization modulation 3D imaging reconstruction based on electro-optical crystals has great development potential and a wide range of application scenarios. In the future, the study of the modulation characteristics of crystals and the high-precision imaging recovery algorithm will be the research focuses for the 3D imaging technology of this system.

## Figures and Tables

**Table 1:** Comparison of SCMOS and ICCD performance parameters.

	Pixel Counts	Pixel Size	Maximum Cable Length	Quantum Efficiency	Readout Noise
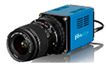	2560 × 2160	6.5 × 6.5 (µm)	10 km	80%	<1.0 e^−^
SCMOS					
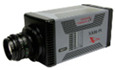	1024 × 1024	12.8 × 12.8 (µm)	5 km	25%	<40 e^−^
ICCD					

**Table 2:** System prototype experimental devices and parameters.

Parameters	Value
Wavelength	532 nm
Pulse Energy	200 mJ
Pulse Duration	7 ns
Repetition Frequency	5 Hz
Resolution	2160 × 2560 pixels
Angle of divergence	0.5°
Crystal clear aperture	9 mm × 9 mm
Modulating voltage	2000 V

## Data Availability

The data that support this study are proprietary in nature and may only be provided with restrictions.
